# Theophylline Use to Prevent Permanent Pacing in the Contemporary Era of Heart Transplantation: The Rotterdam Experience

**DOI:** 10.3389/fcvm.2022.896141

**Published:** 2022-06-24

**Authors:** Claudette Kooij, Tamas Szili-Torok, Stefan Roest, Alina A. Constantinescu, Jasper J. Brugts, Olivier Manintveld, Kadir Caliskan

**Affiliations:** ^1^Department of Cardiology, Thorax Center, Erasmus MC, University Medical Center Rotterdam, Rotterdam, Netherlands; ^2^Erasmus MC Transplant Institute, Erasmus MC, University Medical Center Rotterdam, Rotterdam, Netherlands

**Keywords:** heart transplantation, pacemaker, bradyarrhythmia, theophylline, prevention, indication

## Abstract

**Introduction:**

Sinus node dysfunction and atrioventricular conduction disorders occur increasingly after orthotopic heart transplantation (HTX) due to aging donors and may require permanent pacemaker (PM) implantation. Theophylline has been used in the past in selected cases as an alternative to PM implantation.

**Purpose:**

The aim of this study was to investigate the rate and success of oral theophylline administration after orthotopic heart transplantation preventing permanent PM implantation.

**Methods:**

We included all patients treated with theophylline post HTX due to bradyarrhythmia's in our center from January 1985 to January 2020. Data was obtained retrospectively through electronic patient files. Re-transplants and patients who died within 1 month post HTX were excluded from the analysis.

**Results:**

Of the total of 751 heart transplant recipients, 73 (9,7%) patients (mean age 46 ± 15.2 years; 73% male) were treated with theophylline for bradyarrhythmia's early post HTX. Of these patients, 14 (19%) patients needed a permanent PM during hospitalization and 10(14%) patients stopped using theophylline because of adequate heart rhythm. In the end, 49 (6.5% of the total) patients were discharged with a theophylline (mean maintenance doses of 354 ± 143 mg). At the outpatient clinics, additional 6 (12%) patients needed a PM within 7 months after discharge, with the rest stable sinus rhythm.

**Conclusion:**

In this retrospective data analyses oral theophylline remained a viable alternative to permanent PM implantations in patients post HTX with increased heart rates, facilitating the withdrawal of chronotropic support and avoiding the need of permanent PM implantation.

In the last few decades, early permanent pacemaker (PPM) implantation has been increasingly utilized to treat persistent bradycardia following bradyarrhythmias occurring after transplantation, mainly due to the increasing age of suitable donors in Europe, especially in the Netherlands (1).

There are several disadvantages of PPM implantations such as complications, costs, and delayed hospitalizations related to the procedure within these vulnerable groups of patients. Furthermore, in many patients, the need for pacing vanishes in the months post-HTx (2).

Theophylline is one of the older drug medicines with several pharmacological actions, including bronchial dilatation, diuresis, and chronotropic and dromotropic effect. Therefore, theophylline has been used successfully in the past for the treatment of sick sinus syndrome to increase the heart frequency and avoid PPM implantation (3). The costs of theophylline therapy are minimal, and usually well tolerated. Despite its known use post-HTx, data on its efficacy are scarce.

Therefore, the aim of this study was to investigate the prevalence and efficacy of oral theophylline after orthotopic HTx to prevent PPM implantation.

In this study, data of all consecutive patients who underwent primary HTx at our center between January 1984 and January 2020 were retrospectively collected from the electronic patient records. The demographic and clinical data were collected along with the use and duration of theophylline, dosage, side effects, and early pacemaker implantations. Indications for PPM implantation were categorized into sinus node dysfunction and atrioventricular blockage. Clinically significant bradycardia was defined as a heart rate <60 beats/min. The primary endpoint of this study was the successful discharge of the patient with theophylline without the need of a PPM. Perioperatively, all patients received a temporary pacemaker and isoprenaline intravenously to maintain a heart rate > 100 beats per min in the first 3 days post-HTx. Thereafter, the target heart rate was decreased with 10 beats per min every day until the patient had an intrinsic heart rate of at least 60 bpm with stable hemodynamics. If the heart rate was not sufficient after 10–14 days, theophylline orally with extended release (daily dosage of 200–300 mg) was initiated to taper the isoprenaline intravenously. When theophylline therapy was successful with a stable sinus rhythm of ≥60 bpm and hemodynamics, the patient was discharged with oral theophylline without the need for a PPM. If this was unsuccessful, a PPM implantation was planned in weeks 4 to 6 post-HTX.

Of the total of 751 HTx recipients, 73 (9.7%) patients with a mean age 46 ± 15.2 years, 73% male, were treated with theophylline for bradyarrhythmia post-HTx. Of these patients, 10 (14%) patients stopped using theophylline because of stable sinus rhythm and in 14 (19%) treatment failed followed by a PPM during the hospitalization. Overall, 49 (6.5% of the total) patients were discharged with oral theophylline with a mean maintenance dose of 354 ± 143 mg. At the outpatient clinics, an additional six (12%) patients needed a PPM within the following months (longest: 7 months) post discharge. The prevalence of both theophylline uses (12 patients before and 61 patients after 2000) as well as need of a PPM increased significantly along with increasing donor ages in the past two decades. Figure 1 shows the percentage of theophylline use vs. permanent pacemaker implant at discharge before and after the year 2000, a year in which the baseline immunosuppressive treatment and donor ages changed significantly in our center (1, 4). There were no significant correlations with successful vs. not successful treatment regarding the recipient age (mean 46.6 ± 14.0 vs. 43.8±14.9 years), donor age (mean 40.6 ± 14.7 vs. 42.5 ± 12.7 years), gender, preoperative use of amiodaron (53 vs. 67%), or underlying heart diseases (28 vs. 33 % ischemic heart diseases).

**Figure 1:**
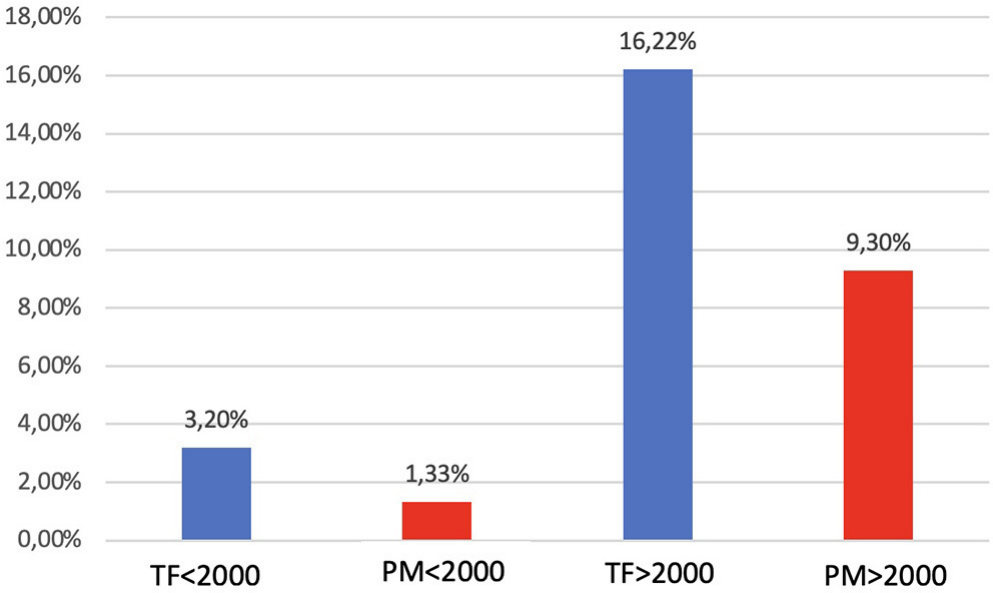
Bar graph showing the percentage of theophylline use vs. permanent pacemaker implant at discharge before and after the year 2000 (*p* = 0.56). TF, theophylline; PM, pacemaker.

The major finding of the present study is that oral theophylline administration early after orthotopic HTx can prevent PPM implantation in a selected group of patients in the contemporary era of HTx. Our single-center experience confirms that the use of theophylline for post-HTx bradyarrhythmias successfully increased the baseline heart rate, facilitated the withdrawal of chronotropic support, and deferred the need for permanent pacing. We recently reported that the most common indication for early PPM implantation was sinus node dysfunction (SND) while atrioventricular block was more frequent in late PM implantation (1). Patients who had an older donor had an increased risk of having a PM implanted both early and late after HT. Unfortunately, the donor ages increased significantly in the last two decades to sustain the declining numbers of suitable heart donors (4). As bradyarrhythmias, especially sinus node dysfunction, usually improve over the weeks to months, the use of theophylline could prevent unnecessary delay of hospital stay post-HTx and save costly PPM implantation besides the low but always existent risk of complications of a PPM. However, randomized controlled trials are needed for a definitive answer in the choice of theophylline vs. early permanent pacemaker implantation.

## Data Availability Statement

The raw data supporting the conclusions of this article will be made available by the authors, without undue reservation.

## Ethics Statement

The studies involving human participants were reviewed and approved by the Institutional Review Board of the Erasmus MC. The patients/participants provided their written informed consent to participate in this study.

## Author Contributions

KC and TS-T contributed to conception and design of the study. CK and KC organized the database, performed the statistical analysis, and wrote the first draft of the manuscript. All authors contributed to manuscript revision, read, and approved the submitted version.

## Conflict of Interest

The authors declare that the research was conducted in the absence of any commercial or financial relationships that could be construed as a potential conflict of interest.

## Publisher's Note

All claims expressed in this article are solely those of the authors and do not necessarily represent those of their affiliated organizations, or those of the publisher, the editors and the reviewers. Any product that may be evaluated in this article, or claim that may be made by its manufacturer, is not guaranteed or endorsed by the publisher.
